# The role of mast cells in the pathogenesis of pain in chronic pancreatitis

**DOI:** 10.1186/1471-230X-5-8

**Published:** 2005-03-03

**Authors:** Willemijntje A Hoogerwerf, Kelly Gondesen, Shu-Yuan Xiao, John H Winston, William D Willis, Pankaj J Pasricha

**Affiliations:** 1 Enteric Neuromuscular Disorders and Pain Laboratory, Division of Gastroenterology and Hepatology, University of Texas Medical Branch, 301 University Boulevard, Galveston, TX 77555-0764, USA; 2 Department of Pathology, University of Texas Medical Branch, 301 University Boulevard, Galveston, TX 77555-1069, USA; 3 Department of Anatomy and Neurosciences, University of Texas Medical Branch, 301 University Boulevard, Galveston, TX 77555-0743, USA

## Abstract

**Background:**

The biological basis of pain in chronic pancreatitis is poorly understood. Mast cells have been implicated in the pathogenesis of pain in other conditions. We hypothesized that mast cells play a role in the pain of chronic pancreatitis.

We examined the association of pain with mast cells in autopsy specimens of patients with painful chronic pancreatitis. We explored our hypothesis further using an experimental model of trinitrobenzene sulfonic acid (TNBS) -induced chronic pancreatitis in both wild type (WT) and mast cell deficient mice (MCDM).

**Methods:**

Archival tissues with histological diagnoses of chronic pancreatitis were identified and clinical records reviewed for presence or absence of reported pain in humans. Mast cells were counted.

The presence of pain was assessed using von Frey Filaments (VFF) to measure abdominal withdrawal responses in both WT and MCDM mice with and without chronic pancreatitis.

**Results:**

Humans with painful chronic pancreatitis demonstrated a 3.5-fold increase in pancreatic mast cells as compared with those with painless chronic pancreatitis.

WT mice with chronic pancreatitis were significantly more sensitive as assessed by VFF pain testing of the abdomen when compared with MCDM.

**Conclusion:**

Humans with painful chronic pancreatitis have an increased number of pancreatic mast cells as compared with those with painless chronic pancreatitis. MCDM are less sensitive to mechanical stimulation of the abdomen after induction of chronic pancreatitis as compared with WT. Mast cells may play an important role in the pathogenesis of pain in chronic pancreatitis.

## Background

Although pain is the presenting symptom of most patients with chronic pancreatitis, its neurobiological basis remains poorly understood [1]. In the past, investigators have focused on the role of anatomical abnormalities such as a strictured pancreatic duct or narrowed intraparenchymal ducts. However, mechanical decompression techniques such as endoscopic stent placement or even surgical pancreatojejunostomy frequently do not provide a permanent solution to the pain [1]. More recently, investigators have begun focusing on the role of neurotransmitters and neurotrophins such as substance P and nerve growth factor with known or suspected roles in nociceptive signaling and/or sensitization and have reported an increased expression of several of them in the pancreas of patients with painful chronic pancreatitis [2]. Mast cells are also increased in both acute and chronic pancreatitis [3,4] but their role in the generation of pain in pancreatitis has not been investigated.

We hypothesized that mast cells are involved in the pathogenesis of pain in chronic pancreatitis. This hypothesis is based on the following observations. First, mast cells have been associated with human conditions in which pain is a predominant symptom. Interstitial cystitis and irritable bowel syndrome are both conditions in which pain is out of proportion to the objective pathological findings [5,6]. In both conditions, an increase in the number of mast cells has been described in the bladder and the colon, respectively [5,6]. Further, mast cells are frequently found in close proximity to nerves in the intestinal mucosa and the bladder [7-9]. This has also been observed in the pancreas – the total number of mast cells was significantly higher in pancreatic tissue from patients with chronic pancreatitis than in the normal pancreatic controls [3]. One of the preferential locations of mast cells was around and within the perineurium of nerve fibers in tissue samples of patients with chronic pancreatitis, suggesting the potential for interactions between mast cells and the nervous system. Lastly, there is evidence for bi-directional functional communication between mast cells and nerves [10-12]. Mast cells can not only release mediators that increase excitability of neurons but in turn, neurotransmitters such as substance P can trigger mast cell degranulation [10]. Mast cells may therefore contribute to the pathogenesis of pain in pancreatitis through degranulation products that can sensitize pancreatic afferent neurons in an ongoing vicious circle of neuronally mediated mast cell degranulation.

Our first aim was to analyze the presence and distribution of mast cells in autopsy specimens of chronic pancreatitis and study the correlation, if any, with historical documentation of pain. We then explored our hypothesis further using an experimental model of trinitrobenzene sulfonic acid (TNBS)-induced chronic pancreatitis in both wild type and Kit^W^/Kit^W-v ^mice, a strain deficient in mast cells (MCDM).

## Methods

### Data collection autopsy study

Autopsy records from the University of Texas Medical Branch from the years 1993 to 2000 were searched electronically for the term "pancreatitis." One-hundred sixty-six patients were identified of which 26 patients carried an autopsy diagnosis of chronic pancreatitis and 140 patients carried a diagnosis of acute pancreatitis. The medical charts from patients with an autopsy diagnosis of chronic pancreatitis were reviewed for documentation of a medical history of chronic pancreatitis. If no such documentation was present in the chart, patients were excluded from the study (12/26). Thus, 14/26 patients with both a documented history and an autopsy based diagnosis of chronic pancreatitis, were included in the study. Patients were categorized as painful chronic pancreatitis (8/26) when they fulfilled one of the following criteria: a documented history of chronic abdominal pain clinically attributed to chronic pancreatitis that required the use of narcotics, and/or frequent admissions for recurrent abdominal pain clinically attributed to chronic pancreatitis, and/or a surgical or endoscopic procedure for refractory abdominal pain clinically attributed to chronic pancreatitis. Patients were categorized as non-painful chronic pancreatitis (6/26) if patients did not fit any of the criteria listed under painful chronic pancreatitis. In addition, the following data were collected: demographic factors (age and race), cause of death, comorbidities, clinical history of pancreatitis, etiology of pancreatitis, diagnostic studies supporting a diagnosis of pancreatitis (amylase, lipase, calcifications on abdominal plain film, CT-scan, ultrasound or ERCP). Human pancreatic control tissue was obtained from 8 arbitrarily chosen patients of whom the autopsy records recorded acute myocardial infarction as the cause of death. Their medical records were reviewed to ensure that they did not have a clinical history of pancreatitis. Therefore there were three categories of patients: one with painful chronic pancreatitis, one with non-painful chronic pancreatitis and non-pancreatitis controls. A pathologist, blinded to the group assignment, verified all histological diagnoses and counted mast cells on a Giemsa stained tissue section (average of 10 high-power randomly chosen (40X) fields per specimen). The protocol was approved by the Institutional Review Board of the University of Texas Medical Branch.

### Mice strains

All mice were purchased from the Jackson Laboratory (Bar Harbor, ME). Male mice were used from the following strain: WBB6F1/j-Kit^W^/Kit^W-v ^(MCDM) and the respective littermate control mouse strain, Kit^W-v ^– +/+ (WT). The mice were 3 months of age at the onset of the experiment with body weights of 25–30 gram.

Experimental protocols involving mice were approved by our Institutional Animal Care and Use Committee (IACUC) in accordance with the guidelines provided by the National Institutes of Health.

### Induction of chronic pancreatitis

**Figure 1:**
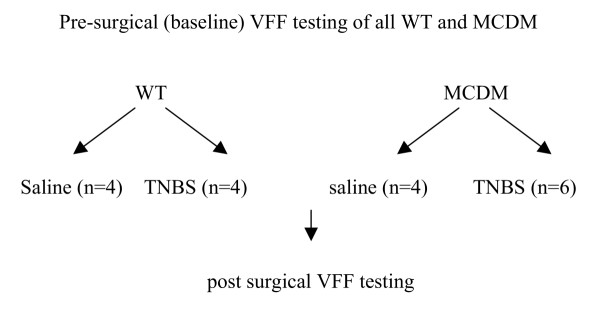
**Experimental design **All mice underwent pre and post surgical VFF testing. For the VFF testing, 4 measures were taken for each mouse. WT and MCDM were randomized to either saline or TNBS perfusion into the pancreatic duct.

Mice were anesthetized with sodium Nembutal (50 mg/kg body weigh, i.p.) Following a midabdominal laparotomy, a canula was introduced into the common pancreato-biliary duct; the duct was ligated proximally and distally to ensure perfusion into the pancreas and prevent entry of the injected substance into the liver or duodenum. 0.1 ml of 1% TNBS in phosphate buffered saline (PBS)-10% ethanol, pH 8, was infused into the pancreas (modified after Puig-Divi [13]). The canula was removed and the abdomen closed. Control mice were treated in the exact same fashion but were perfused with saline instead (Figure 1). Mice were sacrificed 8 weeks after surgery.

### Von Frey Filament (VFF) measurements

VFF hairs consist of a series of filaments of increasing diameter that produce increasing sensations of touch when applied to the skin. When the tip of a fiber of given length and diameter is pressed against the skin, the force of application increases until the fiber bends. After the fiber bends, continued advance creates more bend, but not more force of application. This principle makes it possible to apply a reproducible force to the skin surface. VFF testing is an established behavioral pain assay used to determine mechanical pain thresholds in somatic pain. More recently, VFF testing has been used as a surrogate marker for visceral pain [14,15].

Mice were placed in a cage with a mesh floor and habituated to the environment for 30–60 minutes. Measurements were taken from the abdomen and the plantar surface of both hindpaws over a period of three weeks prior to the surgery and for a total of three weeks after the surgery (Figure 1). VFF filaments of various caliber were applied to the mid-abdomen in ascending order 10 times, each for 1–2 seconds with a 10 second interval. A response was defined as: a) sharp retraction of the abdomen; b) immediate licking or scratching of site of application of hair; or c) jumping. The response frequency was defined as the total number of responses out of 10 applications (expressed as a percentage) to the skin per filament. An investigator blinded to the different treatment groups performed the behavioral testing.

### Pancreatic histology

Fresh specimens of the mouse pancreas were fixed in 10% formaldehyde in PBS pH 7.4 containing 1 mM MgCl_2 _at 4°C overnight. Sections from paraffin-embedded specimens were stained with hematoxyline and eosin and observed under a light microscope. Pathological changes were scored based on a scale described by Tito et al. by a pathologist blinded to the different treatment groups [16].

### Data expression and statistical analysis

Comparisons of the number of mast cells in autopsy specimen were analyzed using the Mann-Whitney U test.

For each behavioral experiment (see figure 1), the average response frequency was calculated as the mean of the mean response frequencies for each mouse (across four measures). The "post-pre response frequency" was calculated by subtracting the pre-surgical average response frequency from the post-surgical average response frequency. To assess the independent effect of TNBS on VFF response (ie. to control for the effect of the surgery itself), the post-pre response frequency for TNBS infusion was compared with the post-pre response frequency for saline infusion. This comparison was performed using analysis of variance for a two-factor experiment with repeated measures on time at each level of force for each type of mice (WT and MCDM). The two factors were induction of pancreatitis or not (TNBS or saline, respectively) and time (pre-surgical or post-surgical). TNBS infusion was considered to have had an independent effect on the VFF response if the post-pre response frequency was greater for TNBS than for saline infusion.

Fisher's least significant difference procedure was used for multiple comparisons of least squares means with Bonferroni adjustment for number of comparisons. All effects and interactions were assessed at the 0.05 level of significance. Data analysis was conducted using PROC MIXED with LSMEANS options in SAS^®^, Release 8.2 [SAS Institute Inc., SAS/STAT^® ^User's Guide, Version 8, Cary, NC: SAS Institute Inc., 1999].

## Results

### Autopsy data

**Figure 2:**
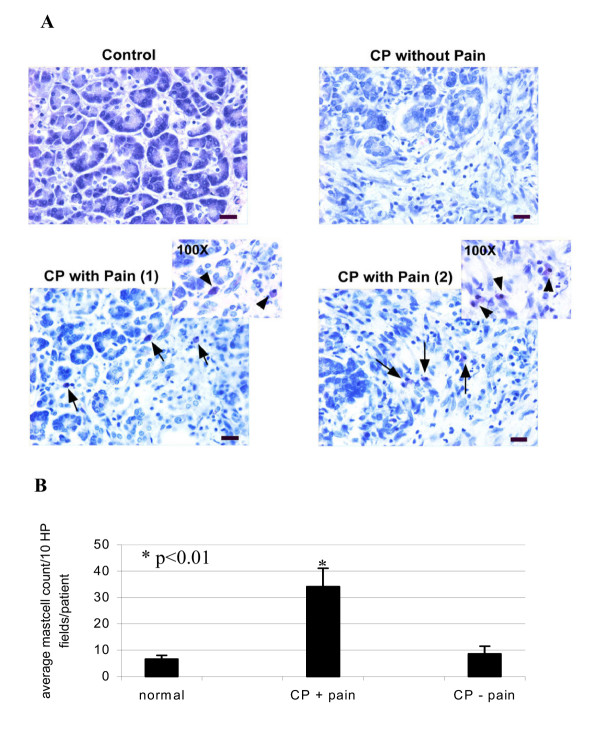
**Mast cell in painful and nonpainful chronic pancreatitis **A Histology of autopsy specimen. Giemsa staining of autopsy specimens of controls and of patients with chronic pancreatitis (CP) both with and without pain. Large images represent 40× magnification while the inserts are 100× magnifications of mast cells. Scale bars represent 20 μM. Arrows point at mast cells. B Autopsy specimens of patients with a history of chronic abdominal pain contained a significantly higher number of mast cells as compared to specimens of patients with chronic pancreatitis without a history of abdominal pain (34.1 +/- 7 vs 8.6 +/- 2.9; P < 0.01) or to control specimens (34.1 +/-7 vs 6.6 +/- 1.4; P < 0.01).

Patient demographics are summarized in Table 1. Alcohol abuse was the most common cause for pancreatitis in both groups. Analysis of our results, using the Mann-Whitney U test, revealed significantly more mast cells in patients with a history of painful chronic pancreatitis (n = 8) when compared to patients with either non-painful chronic pancreatitis (n = 6) (33.8 vs 9.4 average mast cell count/10 high power fields; p < 0.01) or controls (n = 8), (33.8 vs 6.1 average mast cell count/10 high power fields; p < 0.01) (Figure 2). The increased number of mast cells in patients with painful pancreatitis was noted predominantly in interstitial areas and, to a lesser degree, in the periacinar space.

### Withdrawal responses to mechanical stimulation of the abdomen and paw after induction of chronic pancreatitis

**Figure 3:**
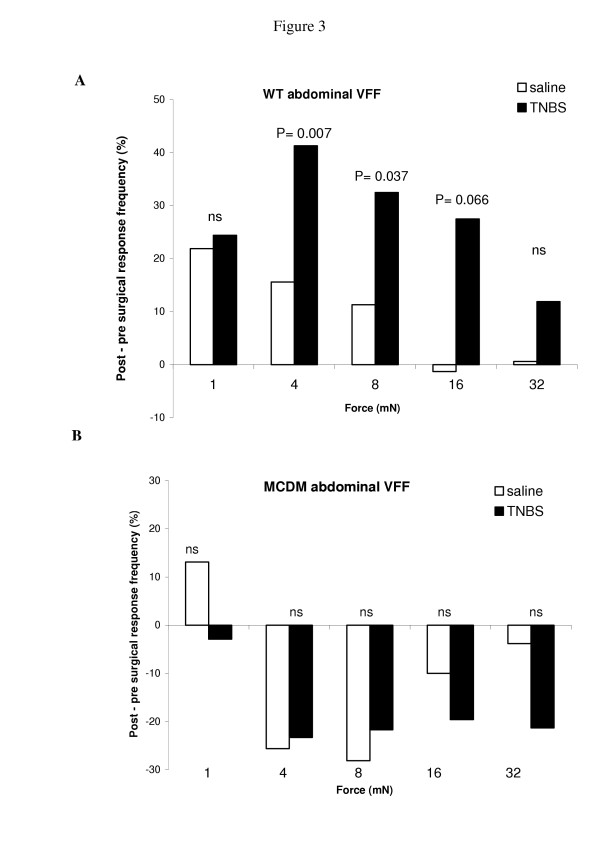
**Abdominal VFF WT and MCDM **Post-pre response frequency was calculated by subtracting the average pre-surgical response frequency from the average post-surgical response frequency for WT mice (A) and MCDM (B). P-values are for the comparison of the post-pre response frequency for TNBS infusion versus post-pre response frequency for saline infusion.

**Figure 4:**
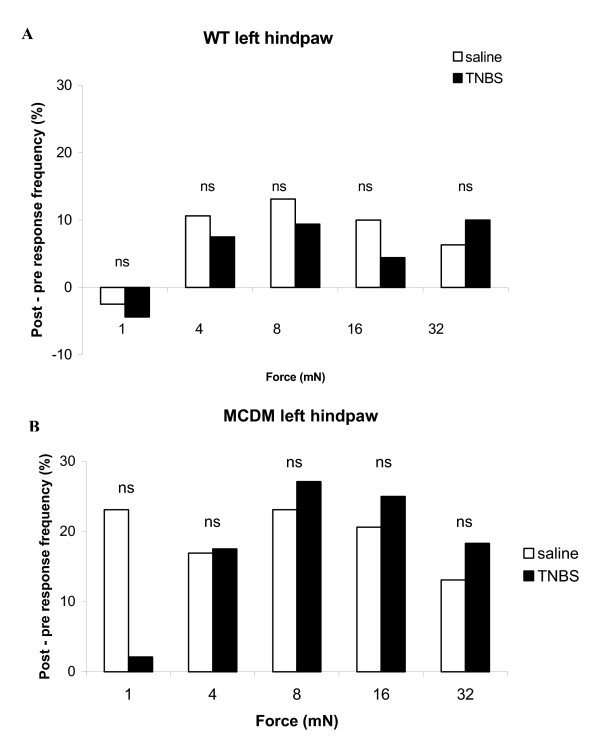
**Left Hindpaw VFF WT and MCDM **Post-pre response frequency was calculated by subtracting the average pre-surgical response frequency from the average post-surgical response frequency for WT mice (A) and MCDM (B). P-values are for the comparison of the post-pre response frequency for TNBS infusion versus post-pre response frequency for saline infusion.

Figure 3 shows the post-pre surgical response frequency for both WT and MCDM. TNBS had a significant independent effect on abdominal VFF response in WT mice at the force levels 4 and 8 mN (p = 0.007 and 0.037, respectively) (Figure 3A). There was a trend towards a significant effect at the force level of 16 mN (p = 0.066). In contrast, for MCDM, TNBS had no significant effect on abdominal VFF response at any force level (Figure 3B). There was no significant TNBS effect on VFF response in the left hindpaw for either WT mice or MCDM (Figure 4). Data not shown for the right hindpaw.

### Histological analysis of mice pancreas following TNBS injection

**Figure 5:**
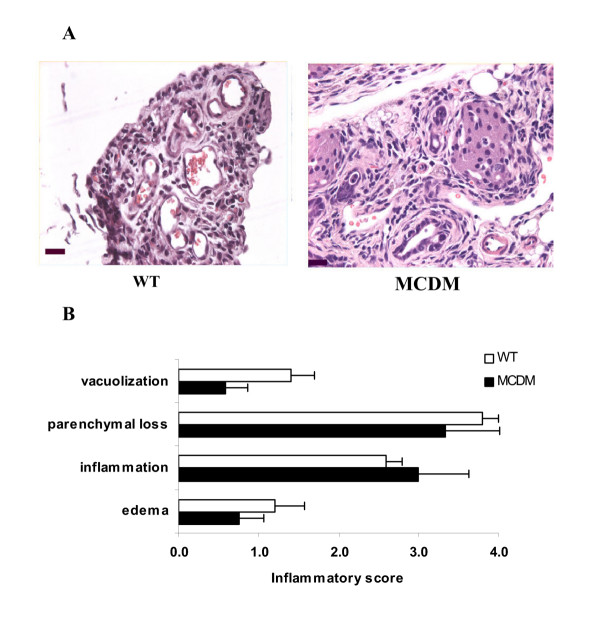
**Histology (H&E) of mice with chronic pancreatitis **A H&E staining of the pancreas of WT and MCDM 4 weeks after infusion of TNBS into the pancreatic duct. Images represent 40× magnification; scale bars represent 20 μM. Fibrosis and inflammatory infiltrates are present. B Average histological scores for WT and MCDM. There is no significant difference in histological scores between WT and MCDM (P = NS).

**Figure 6:**
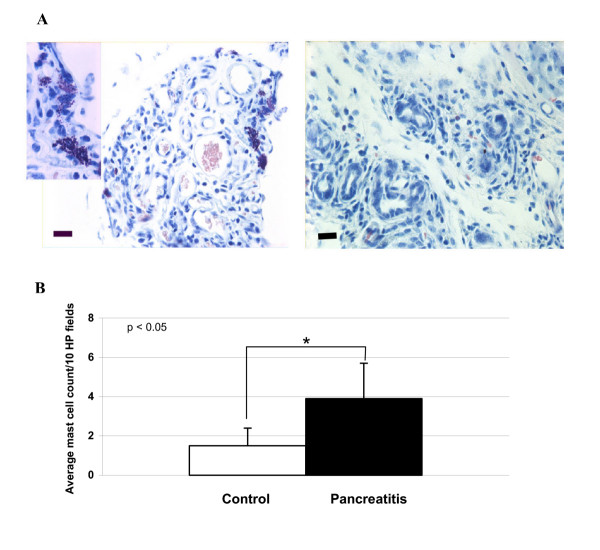
**Histology (Giemsa) of mice with chronic pancreatitis **A Giemsa staining of the pancreas of WT and MCDM 4 weeks after infusion of TNBS into the pancreatic duct. Large images represent 40× magnification while the inserts are 100× magnifications of mast cells. Scale bars represent 20 μM. B Significantly more mast cells are present in the pancreatic tissue of mice treated with TNBS when compared with saline treated controls (* P < 0.05).

Pancreatic histology confirmed the presence of chronic pancreatitis in both WT and MCDM with marked fibrosis, inflammatory infiltrates and ductular proliferation mimicking changes seen in human chronic pancreatitis (Figure 5A). The pancreas of saline treated controls was normal. There was no significant difference in the overall inflammatory scores between the WT and MCDM (Figure 5B). An increased number of mast cells were counted in WT mice with chronic pancreatitis compared to saline treated controls (5.6 vs 1.5; p = 0.05) (Figure 6). As to be expected, no mast cells were present in pancreas of MCDM.

## Discussion

Chronic pancreatitis has been defined as a continuing inflammatory disease of the pancreas characterized by irreversible morphologic changes that typically cause pain and/or permanent loss of function [17]. The pathogenesis of pain in this condition remains to be satisfactorily established. We examined the association, if any, of pain with mast cells as quantified in autopsy specimens of patients with a history of painful and non-painful chronic pancreatitis and normal controls. Significantly more mast cells were present in pancreatic tissue from patients with a history of painful chronic pancreatitis, indicating an association with this condition and a potential role for these cells in the pathogenesis of pain in painful chronic pancreatitis.

There are clearly limitations to a retrospective, autopsy-based study such as the one we report here. For instance, we do not know whether pain was present at the time of death and there was incomplete information on the different patterns of pain. Also, our findings pertain mainly to patients with a history of alcoholic pancreatitis. Nevertheless, our findings do suggest an association of painful chronic pancreatitis with an increased number of mast cells. This observation provided the rationale for further experimental testing, which we performed in mice. We first developed a model of chronic pancreatitis in mice following a modified protocol first described by Puig- Divi *et al*. [13]. Histological changes consisted of periductal and lobular fibrosis, duct stenosis, chronic inflammatory cell infiltrates, and gland atrophy, mimicking features of chronic pancreatitis in humans. Significantly more mast cells were present in WT mice with chronic pancreatitis, adding to the validity of this model for use in studies on the role of mast cells in pancreatitis. Both WT and MCDM developed histological changes consistent with chronic pancreatitis, indicating that the elimination of mast cells did not modulate the animals' ability to mount an inflammatory response. Therefore, any changes observed in pain behavior are unlikely to stem from differences in underlying inflammation.

Next we determined whether this mouse model could be used to study behavioral differences associated with chronic pancreatitis. The assessment of spontaneous pain in a visceral organ presents significant difficulties. We have used a behavioral method to assess this, which relies on the association of visceral pain with sensitization of somatic regions of the body that share segmental innervation at the level of the spinal cord (referred pain). This somatic sensitization can be quantified using VFF to stimulate the somatotopically appropriate abdominal region and measuring the abdominal withdrawal response. Thus, VFF testing of the anterior abdominal wall can be used as a surrogate marker for visceral pain. Although this is the first time that this technique has been used for the measurement of referred visceral hyperalgesia in a mouse model of chronic pancreatitis, this method has previously been described and validated to assess the severity of referred visceral pain for models of colonic hypersensitivity [14] as well as rat models of acute necrotizing pancreatitis [15] and chronic pancreatitis [18]. The abdominal VFF response was compared to the hind paw response to assess the specificity of the interventions to the pancreas. TNBS treated mice, but not the saline control, developed increased abdominal wall withdrawal responses to VFF testing when compared to baseline, suggesting the development of force-dependent referred hyperalgesia of the abdominal wall in WT mice. There was no evidence of referred hyperalgesia in the hindpaws, suggesting that the measured effect on abdominal withdrawal is specific for an intra-abdominal origin of the pain. Vera-Portocarrero et al. previously described similar findings, increased withdrawal frequency after VFF stimulation to the abdominal area, in a rat model of chronic pancreatitis [18]. These behavioral changes were abrogated by morphine. Rats that demonstrated behavioral changes also expressed increased substance P expression in the nociceptive layers of the spinal cord, suggestive of central nociceptive changes.

**Figure 7:**
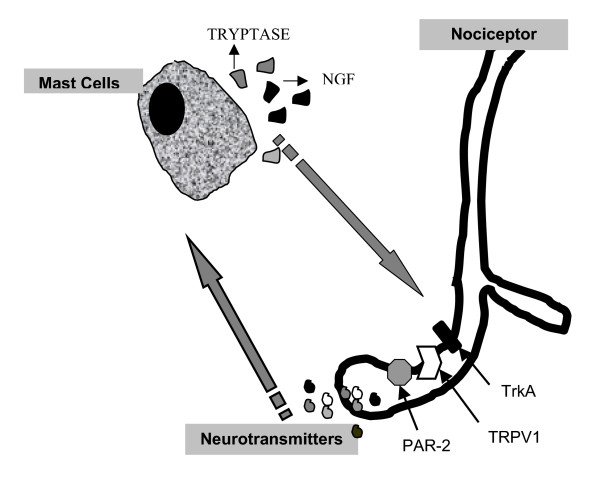
**Proposed involvement of mast cells in nociceptive signaling in pancreatitis **In pancreatitis, mast cells may migrate to sites of inflammation, in response to release of mast cell chemoattractants. Mast cell degranulation products may modulate neurotransmission directly by activating proalgesic receptors and channels such as trka (NGF), TRPV1 (NGF) and PAR-2 (tryptase and trypsin).

Mast cells produce a variety of degranulation products in the setting of inflammation that may activate and/or sensitize primary nociceptive neurons. The neurotrophin growth factor (NGF) is one such product [19-22]. NGF is released in the setting of inflammation and can not only function as a chemoattractant for other mast cells, but it can also trigger mast cell degranulation [23]. We are speculating that NGF production in the inflamed pancreas is responsible for plastic changes in the sensory neurons by activating proalgesic receptors and channels such as the NGF receptor tyrosine kinase A (TrkA) and Transient Receptor Potential Family V receptor 1 (TRPV1; previously known as VR1) thereby contributing to the generation of pain [24-26]. Similarly, other mast cell degranulation products such as tryptase and histamine are capable of modulating neuronal function [27-32]. Tryptase may directly activate the proteinase-activated receptor-2 (PAR-2), a G-protein coupled receptor expressed by pancreatic nerves, important in the pathogenesis of pain in pancreatitis [33,34]. Although the role for mast cells in the mediation of visceral nociceptive signaling needs to be explored further, we speculate that mast cell products released in pancreatitis, contribute to the development of pain by direct effects on nociceptors located on pancreatic afferent neurons (Figure 7).

However, before concluding a definite role for mast cells from our experimental data, it should be noted that MCDM carry a spontaneous mutation for tyrosine kinase receptor c-kit which not only produces a deficiency of mast cells but may have an independent effect on the function of sensory neurons, which are known to express it [35]. Therefore, it remains to be determined whether the detected differences in nociceptive responses is due to the absence of mast cells per se or a yet unknown change in the responsiveness of sensory neurons due to a congenital lack of the c-kit receptor. Reconstitution of mast cells into the MCDM mice should restore their nociceptive responses close to the wild type phenotype.

## Conclusion

Our data should increase awareness of the importance of mast cells in the pathogenesis of painful inflammatory conditions such as chronic pancreatitis and encourage experimental studies for further testing of this hypothesis.

## Abbreviations

TNBS – trinitrobenzene sulfonic acid, WT – wild type, MCDM – mast cell deficient mice, VFF – von Frey Filaments, IACUC – Institutional Animal Care and Use Committee, PBS – phosphate buffered saline, NGF – neurotrophin growth factor, TrkA – tyrosine kinase A, TRPV1 – Transient Receptor Potential Family V receptor 1 (previously known as VR1), PAR-2 – proteinase-activated receptor-2

## Competing interests

The author(s) declare that they have no competing interests.

## Authors' contributions

WAH conceived of the study, participated in its design and coordination and wrote the manuscript. KG completed the behavioral studies. SYX completed the histological analyses and mast cell counts. JHW provided technical assistance with the surgical procedures and participated in the data analyses and review of the manuscript. WDW provided support for the behavioral studies as well as their analyses. PJP provided financial support and participated in the review of the manuscript.

## Pre-publication history

The pre-publication history for this paper can be accessed here:



## Figures and Tables

**Table 1:** Patient demographics

A Patients with chronic pancreatitis and pain								
	Patient 1	Patient 2	Patient 3	Patient 4	Patient 5	Patient 6	Patient 7	Patient 8

Age	54	58	38	63	61	65	38	73
Race/gender	B-M	C-M	LA-M	C-M	C-F	B-M	C-M	C-M
Cause of death	arrhythmia	AMI	Cardio-myopathy	pneumonia/ESLD	liver-failure	CAD/cachexia	Gibleed/ARDS	PE
Comorbidities	ESRD/CP/HCV	COPD/CP	CP/sp Puestow	cirrhosis/COPD/CP	HTN/CP	HTN/CP	CP/sp Puestow	COPD/CAD/CP/Laryngeal ca
CP etiology	ETOH	ETOH	chronic cholelithiasis	ETOH	ETOH	ETOH	ETOH	ETOH
CP imaging	CT	CT/ERCP	CT/ERCP	CT	CT/ERCP	CT/ERCP	CT/ERCP	CT
Enzymes	no	no	yes	no	no	no	yes	yes
Insulin	no	no	yes	no	no	yes	yes	no
Narcotics	vicodin	vicodin	vicodin	vicodin	vicodin	darvocet	vicodin	vicodin

B Patients with chronic pancreatitis without pain								
		
	Patient 1	Patient 2	Patient 3	Patient 4	Patient 5	Patient 6		
		
Age	56	72	56	60	45	40		
Race/gender	C-M	C-F	C-M	C-M	C-F	C-F		
Cause of death	AMI	sepsis	AMI	pneumonia	sepsis	unknown		
Comorbidities	CP	CP	IDDM/CP	CAD/CM/IDDM/CP	HIV/asthma/HTN	CP		
CP etiology	ETOH	ETOH	idiopathic	idiopathic	ETOH	ETOH		
CP imaging	CT/ERCP	RUQ-US	CT	CT	CT	CT		
Enzymes	yes	yes	no	yes	no	yes		
Insulin	yes	no	yes	yes	no	yes		
		
C Control group								
		
	Control 1	Control 2	Control 3	Control 4	Control 5	Control 6		
		
Age	56	56	73	51	53	76		
Race/gender	C-M	LA-F	C-F	LA-M	C-M	C-F		
Cause of death	AMI	AMI	arrhythmia	AMI	PE	AMI		
Comorbidities	HTN	CAD	CAD/HTN/DM	DM	CAD	CAD/HTN/DM		

C = caucasian; LA = latin-american; B = black; M = male; F = female; ESRD = end stage renal disease; HCV = hepatitis C virus; COPD = chronic obstructive pulmonary disease; AMI = acute myocardial infarction; ESLD = end stage liver disease; HTN = hypertension; CAD = coronary artery disease; ca = cancer; ETOH = alcohol; ERCP = endoscopic retrograde cholangiopancreatogram; IDDM = insulin-dependent diabetes mellitus; CM = cardiomyopathy; HIV = human immunodeficiency virus
